# Effects of the COVID-19 associated United Kingdom lockdown on physical activity in older adults at high risk of cardiovascular disease: a mixed methods perspective from the MedEx-UK multicenter trial

**DOI:** 10.3389/fpubh.2024.1371453

**Published:** 2024-05-09

**Authors:** Richard J. Elsworthy, Stephanie T. Jong, Sarah Hanson, Oliver M. Shannon, Amy Jennings, Rachel Gillings, Mario Siervo, Michael Hornberger, Wendy Hardeman, John C. Mathers, Anne-Marie Minihane, Sarah Aldred

**Affiliations:** ^1^School of Sport, Exercise and Rehabilitation Sciences, College of Life and Environmental Sciences, University of Birmingham, Birmingham, United Kingdom; ^2^School of Health Sciences, Faculty of Medicine and Health Sciences, University of East Anglia, Norwich, United Kingdom; ^3^Human Nutrition and Exercise Research Centre, Centre for Healthier Lives, Population Health Sciences Institute, Newcastle University, Newcastle upon Tyne, United Kingdom; ^4^The Institute for Global Food Security, School of Biological Sciences, Queen's University Belfast, Northern Ireland, United Kingdom; ^5^Norwich Medical School, Faculty of Medicine and Health Sciences, University of East Anglia, Norwich, United Kingdom; ^6^School of Population Health, Curtin University, Perth, WA, Australia

**Keywords:** physical activity, cardiovascular disease, coronavirus, lockdown, mixed methods, adiposity

## Abstract

**Introduction:**

Physical inactivity and sedentary behaviour are linked to increased risk of cardiovascular disease, infections and dementia, as well as placing a significant economic burden on healthcare systems. The implementation of COVID-19 pandemic lockdown measures aimed at reducing virus transmission posed challenges to the opportunity to be physically active. This study investigates how the first UK COVID-19 lockdown affected objectively measured physical activity in older adults at higher risk of cardiovascular disease.

**Methods:**

We studied 48 individuals aged 55-74 years (81.3% female) with self-reported PA levels < 90 min/week and a QRISK2 score ≥ 10 (indicative of a ≥ 10% risk of a major cardiovascular event in the next 10 years) without mild cognitive impairment or dementia. Physical activity data was collected using objective wrist-based activity monitors and analysed across three time periods, usual activity (pre-pandemic), the precautionary phase when the UK began advising on limiting social contact and finally during the first UK lockdown period was collected (27 January 2020 and 07 June 2020). Data was analysed using linear mixed effects model was used to investigate PA levels over the measured 12-week period. Effects of BMI, age, deprivation score and baseline PA levels on PA across the three measurement periods were also examined. Focus-group and individual interviews were conducted, and data were thematically analysed.

**Results:**

Average daily step count (−34% lower, *p* < 0.001) and active energy expenditure (−26% lower, *p* < 0.001) were significantly lower during the precautionary period compared with the usual activity period. Physical activity remained low during the UK lockdown period. Participants with a lower BMI engaged in significantly more (+45% higher daily steps *p* < 0.001) physical activity and those over 70 years old were more physically active than those under 70 years across the 12-week period (+23% higher daily steps *p* < 0.007). The risk of COVID-19 infection and restrictions because of lockdown measures meant some individuals had to find alternative methods to staying physical active. Participants described a lack of access to facilities and concerns over health related to COVID-19 as barriers to engaging in physical activity during lockdown. For some, this resulted in a shift towards less structured activities such as gardening or going for a walk.

**Discussion:**

The data presented shows that lockdown measures during the COVID-19 pandemic significantly reduced physical activity among older individuals at risk of cardiovascular disease, particularly those with a higher body mass index. To support this population group in staying active during future lockdowns, a multifaceted strategy is needed, emphasizing psychosocial benefits and home-based physical activity. The MedEx-UK study was pre-registered with ClinicalTrials.gov (NCT03673722).

## Introduction

In the United Kingdom, physical inactivity is estimated to cost the NHS around £455 million per year (1), representing a considerable economic burden. Regular low-to-moderate intensity physical activity (PA) is a modifiable risk factor that reduces cardiovascular disease risk, improves immune function, and may reduce risk of upper respiratory tract infections (2, 3) but protection against such infections is uncertain (4). Both low PA and high sedentary time present significant risk for future dementia development, through direct effects (5) or associated cardiometabolic comorbidity (6, 7). For example, skeletal muscle mass and function are reduced with as little as 2 days of inactivity (8), whilst whole-body metabolism and adipose tissue function are perturbed following 7 days of reduced PA (9). Acute reduction of PA (3–14 days) inhibits insulin sensitivity and glycaemic control and increases adiposity (10). Likewise, individuals with reduced PA and increased sedentary time are likely to experience worse physical functioning and poorer quality of life (11, 12). This is of particular importance for older adults (≥60 years) who are at greater risk of risk of all-cause and cardiovascular mortality, cancer, cognitive decline, and events associated with physical frailty such as fractures, recurrent falls, and disability (13). Low habitual PA can also negatively impact mental health, increasing the risk of anxiety and depression, and further increasing dementia risk (14). This is of particular importance as there is growth of the ageing population relative to the working-age population, alongside increased multimorbidity, which will ultimately place further strain on the national health service (15). Promoting PA can positively impact ‘healthy ageing’ and therefore, it is crucial that strategies to promote moving more and sitting less in older adults are implemented to lower disease burden and maintain quality of life (16). However, several unprecedented challenges to PA emerged during early 2020, with the worldwide spread of Severe Acute Respiratory Syndrome Coronavirus-2 (SARS-CoV-2), which resulted in the coronavirus 2019 (COVID-19) pandemic. The United Kingdom national ‘lockdown’ measures in response to the COVID-19 pandemic were designed to reduce person-to-person transmission of the virus and to minimise infection rates. These included limiting outdoor activity, maintaining a 2-m gap between individuals from different households, avoiding non-essential travel, working from home, and closures of schools, universities, non-essential shops, and other businesses. Further guidelines were stipulated for individuals with pre-existing medical conditions, who were at enhanced risk of serious illness and death from COVID-19. Evidence of a significant reduction in PA, increased sedentary time and poorer mental wellbeing in healthy cohorts have been identified across international research (17–22). However, much of this research relied upon self-reported measurements of PA that are prone to misreporting (23).

Despite widespread reductions in PA across different population groups, there is an indication that the impact of COVID-19-related lockdown may have differed across different demographic groups. Individuals who were living alone, had higher pre-pandemic PA levels, a lower income or experienced a loss of employment experienced greater reductions in PA because of the pandemic (24). Contradicting evidence suggested, older United Kingdom adults reported maintenance or even increases in PA from pre-pandemic levels but this benefit was linked to greater periods of sedentary behaviour (25) and, overall, PA levels were lower. Those with a greater body mass index (BMI) reported greater falls in PA as a result of the pandemic in the United Kingdom (26). However, the impact of COVID-19 restrictions on PA amongst older adults at a greater risk of cardiovascular disease has not been investigated and much of the available data on activity levels during COVID-19 restrictions relies on subjective data collection. Individuals at greater risk of cardiovascular disease may be particularly susceptible to negative impacts of restrictions on daily living, further driving the progression if disease burden and increasing the risk of mortality from COVID-19 (27). Even acute periods of physical inactivity can significantly impact cardiometabolic health (28).

The current study examines how the first United Kingdom lockdown (27 January 2020 and 07 June 2020) affected objectively measured PA, in older adults, who were at higher risk of cardiovascular disease (29). This data were collected using wrist-worn activity monitors (Vivosmart 3, Garmin, Hampshire, United Kingdom). Quantitative data were used to generate an understanding of average daily step counts and active energy expenditure. Qualitative data were also collected to provide unique insight into individual perspectives and experiences of how the COVID-19 pandemic influenced PA. Therefore, this data will seek to contextualise participants’ experiences during lockdown and detail the perceived barriers and facilitators to being physically active. To achieve this, we used data from participants in the MedEx-UK study. This was a randomised feasibility study combined with a mixed methods process evaluation which ran throughout the first year of the COVID 19 pandemic. The current study seeks to demonstrate the impact of lockdown on PA behaviour, and better understand facilitators and barriers to PA.

## Materials and methods

### Study population and design

The current study is an explanatory design using mixed methods data: quantitative data from the feasibility RCT, and qualitative data from the MedEx-UK process evaluation, which was embedded in the RCT. Data were collected from a subset of participants who had available data during the weeks surrounding the first United Kingdom lockdown (see Object assessment of physical activity for dates) in the MedEx-UK multicentre feasibility RCT. The full MedEx-UK trial included 104 individuals (74% female, 57–76 years), recruited via general practises and direct-to-public advertisement in three sites in the United Kingdom: Birmingham, Newcastle, and Norwich. To be eligible, participants were required to have self-reported PA levels <90 min/week, a Mediterranean diet score of <9 on a 14-point scale (30), a QRISK2 score ≥ 10 (indicative of a ≥ 10% risk of a major cardiovascular event in the next 10 years) and to be free of mild cognitive impairment and dementia. Participants were recruited to take part in this study through primary care and via direct-to-public advertisements (e.g., targeted social media advertisements and posters). Participants received modest financial remuneration. In the study intervention groups, this comprised £30/week for 24 weeks to support purchase of food throughout the study. Participants in the control group were given vouchers worth £240 (equivalent to £10/week enrolled in the study) on completion. Full description of the study protocol has been published elsewhere (31). In the analysis, 48 participants who had available data for three specific time periods were included, i.e., (1) usual activity (pre-pandemic), (2) precautionary phase when the United Kingdom began advising on limiting social contact, and (3) the first United Kingdom lockdown period. Data were analysed to investigate how PA levels were impacted by United Kingdom government restrictions in response to the COVID-19 pandemic. MedEx-UK received NHS REC and HRA approval (18/NI/0191), and participants provided informed consent.

### Objective assessment of physical activity

**Figure 1:**
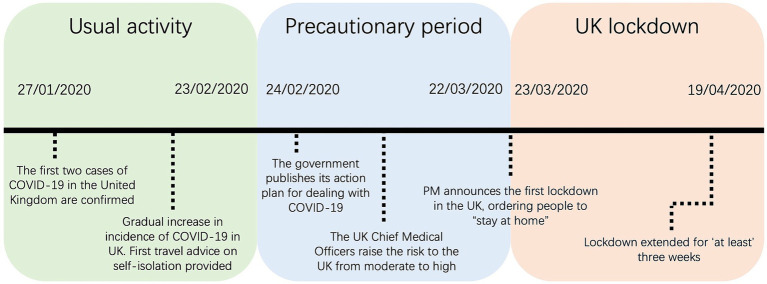
Timeline of the significant events during the COVID-19 pandemic corresponding to dates used within the study. Usual activity (27/01/20–23/2/20), precautionary (24/2/20–22/3/20), and lockdown (23/3/20–19/4/20).

Participants wore a commercially available activity monitor (Vivosmart 3, Garmin, Hampshire, United Kingdom) throughout intervention period. Information on using the device can be found at the following link https://www8.garmin.com/manuals/webhelp/vivosmart3/EN-US/vivosmart_3_OM_EN-US.pdf from the manufacturer. Participants were asked to wear the tracker on their non-dominant wrist throughout the study intervention period. They were instructed to charge the device overnight every 3–4 days, but otherwise were encouraged to wear it at all times (including when carrying out water-based activities). The activity monitors were set to show the time and date, preventing the participants from accessing any activity-based data to minimise the influence of data feedback on behaviour (32). No GPS was active during the study. The participant’s age, height, and weight were entered when setting up the device according to the manufacturer’s guidelines. Daily step count and active energy expenditure (Kcal) between 27 January 2020 and 07 June 2020 was collected. ‘Active Kcals’ corresponds to energy expenditure through movement and activity above predicted basal metabolic rate. These three distinct periods were defined to allow exploration of the effects of the United Kingdom lockdown and potential anticipation of the lockdown (during a precautionary phase) on PA levels compared with usual behaviour and captures the transition in United Kingdom restrictions from living as usual through to full lockdown measures. The 12 weeks were categorised into the 3 × 4-week predetermined periods, namely: usual activity (27/01/20–23/2/20), precautionary (24/2/20–22/3/20), and lockdown (23/3/20–19/4/20). These dates correspond to phases of the first United Kingdom lockdown. Average daily step count was calculated in individuals with 4 or more days of continuous wear, for at least 1 week, within each of the predetermined periods (see Figure 1).

### Assessment of other variables

Height and weight were assessed during pre-trial screening, BMI was calculated, and participants categorised as healthy weight (18.5–24.9 kg/m^2^), overweight (25.0–29.9 kg/m^2^), and living with obesity (30.0–39.9 kg/m^2^). Age at baseline was dichotomised at 70 years. Participant postcodes were used to calculate the Index of Multiple Deprivation using an online calculator^1^ that uses information on seven-sub domains of deprivation (income, employment, education, skill and training, health and disability, crime, barriers to housing and services, and living environment) to calculate an area-based index. For analysis, participants were dichotomised into higher (deciles 1–5) and lower (deciles 6–10) levels of deprivation, this is in line with previous work using the Index of Multiple Deprivation (33). Median step count during the ‘usual activity’ period was used to categorise participants into ‘higher’ or ‘lower’ activity group at baseline.

### Qualitative data collection

The qualitative data were collected for the MedEx-UK mixed methods process evaluation. The research questions for this were underpinned by constructivism: giving voice to participants to share their accounts of their attitudes to, and their experiences of, implementation of behaviour change related to PA and eating, including contextual factors (e.g., different locations, impact of lockdown). Semi-structured focus groups were used to gain an understanding of experiences of participating in the study (34). The interview guide was informed by the theoretical framework of acceptability (35), as well as questions about the feasibility of behaviour change, and the Capability-Opportunity-Motivation-Behaviour Model (COM-B model) (4). The final questions within the interview guide were relevant to the impact of COVID-19. Noteworthy, participants discussed implications of COVID-19 throughout the focus groups.

All MedEx-UK participants were invited via email to take part in focus groups and those who expressed interest were provided a link to attend the online focus group. Participants were not asked their reasons for non-participation. For participants who could not meet with the focus group at the time planned, and for one participant who preferred a one-to-one interview, individual interviews were conducted. All participants gave informed consented prior to the data collection. An experienced qualitative researcher, practised in the field of health behaviours (STJ), conducted all focus groups and interviews. STJ was unknown to the participants and at the start of the focus group or interview, participants were informed that STJ had not been involved in either the design or delivery of the intervention.

Fourteen semi-structured focus groups were conducted (across Birmingham, Newcastle, and Norwich), alongside four individual interviews. Focus groups were conducted between April and September 2020, after the United Kingdom lockdown was introduced during March–June 2020, and restrictions were re-imposed during September–October 2020. Focus groups lasted between 43 min to just over 2 hours and varied between two and six participants. Participants were in focus groups with the participants with whom they had been in the group-based intervention. Interviews lasted between 35 and 54 min. Focus groups and interviews were held online via videoconferencing software informed by previous research on using videoconferencing for qualitative data collection (36). The online format meant that participants were at home for data collection. One individual interview was held via telephone due to participant technical difficulties. Remote focus groups/interviews were used due to SARS-CoV-2 pandemic restrictions on travel and contact, and the dispersed geographical locations of participants.

### Statistical analysis

All analyses were conducted using GraphPad PRISM Version 10.0.3 (GraphPad, San Diego, CA, United States), and an alpha level of *p* < 0.05 considered for determination of statistical significance. Baseline characteristics are reported as mean ± standard deviation, and average step count per day as adjusted mean ± standard deviation, unless stated otherwise. Characteristics of the included participants were compared with the non-included MedEx-UK participants using one-way ANOVA. A linear mixed effects model was used to investigate PA levels over the measured 12-week period. For significant interaction effects, *post-hoc* analysis with a Bonferroni adjustment for multiple comparisons was conducted. Effects of BMI, age, deprivation score, and baseline PA levels on PA across the three measurement periods were also examined. Additional variables (i.e., BMI, age, deprivation score, and baseline PA) were analysed using independent samples *t*-tests. Normality and homogeneity of variance assumptions were met for all analyses.

### Qualitative data analysis

Audio recordings were transcribed verbatim and uploaded and managed in NVivo 12 (QSR International Pty Ltd., 2018). Data were analysed by STJ following Braun and Clarke’s thematic analysis six phase process (37, 38). Initially, STJ and SH deductively double-coded a subset of five transcripts against the core components of the MRC process evaluation guidance (39) as well as the COM-B model (4) for influences on behaviour (change); and then we took a more inductive approach. Throughout analysis, STJ continually reflected upon their influence on the interpretation of data, particularly around beliefs about health and PA. Whilst acknowledging the reflective influence of STJ’s interpretations, the focus was on participants’ accounts of their experience and attitudes to behaviour change. The quotes used in this paper typify participant contributions to focus group discussions and exemplify a theme of ‘the impact of SARS-CoV-2’, linked to ‘context’ within the MRC process evaluation guidance (39). The qualitative data were integrated with the quantitative findings to illuminate, and deepen our understanding of, the objective PA results (40).

## Results

Results presented include mixed methods data synthesis and will report quantitative and qualitative data separately. First, quantitative data including participant characteristics and objectively measured PA will be presented, this will be followed by qualitative synthesis.

### Participant characteristics

From the MedEx-UK RCT, a total of 48 individuals were eligible for inclusion in the quantitative analysis having steps and energy expenditure data available at the time of the first United Kingdom lockdown (Jan–June 2020). Baseline characteristics of included participants are compared with those of the other 56 participants without available data in Table 1. No significant differences were found between groups (*p* > 0.05). Data presented herein, are independent of assigned treatment group for the MedEx-UK RCT unless stated.

**Table 1:** Demographics of the group included for quantitative analysis compared to the remaining MedEx-UK RCT.

	Included (*n* = 48)	Excluded (*n* = 60)
Age (years)	67.65 ± 4.13	66.88 ± 4.94
% Female	81.3	73.3
≥70 years (*n*)	19	17
Weight (kg)	79.06 ± 12.87	78.95 ± 13.32
BMI (kg/m^2^)	29.07 ± 4.87	28.37 ± 3.91
Deprivation score	17,123 ± 9,177	17,578 ± 8,403
QRISK score (%)	16.79 ± 5.34	16.37 ± 5.32

### Changes in physical activity across time periods

**Figure 2:**
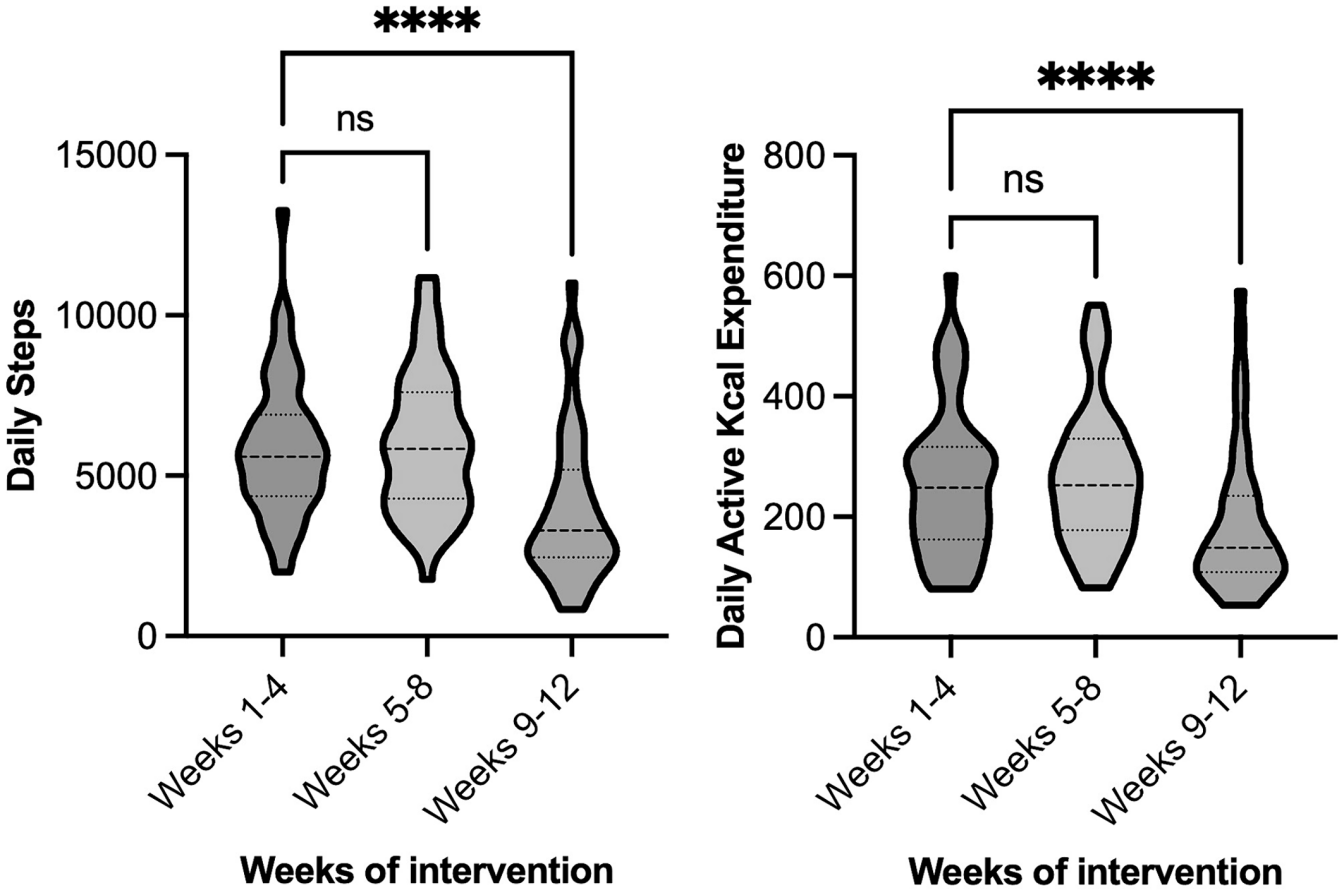
Physical activity of all participants during the 12-week measurement period. The 12 weeks of continuous observation were categorised into the 3 × 4-week predetermined periods, namely: usual activity (27/01/20–23/2/20), precautionary (24/2/20–22/3/20), and lockdown (23/3/20–19/4/20). Left chart—Average daily steps, Right chart—Average daily active kcal expenditure. ^****^Significantly different to matched group, *p* < 0.05.

Figure 2 shows the mean number of steps per day and active kcal expenditure each week throughout the 12 weeks of measurement. Mean daily PA (measured as steps and as active kcal expenditure) were similar during the 4 weeks of usual activity and the 4 weeks precautionary phase. However, both daily step count and active kcal expenditure fell sharply during the lockdown period. This occurred irrespective of intervention group. Across all participants, there was a significant reduction in the number of steps per day from the usual activity (5,897 ± 2,189) to lockdown (−33% reduction, 3,977 ± 2,378, *p* < 0.001), and precautionary (6,026 ± 2,213) to lockdown periods (−34% reduction, *p* < 0.001). There was also a significant reduction in active kcal expenditure per day from the usual activity (283 ± 167) to lockdown (−28% reduction, 203 ± 134, *p* < 0.001), and precautionary (275 ± 128) to lockdown periods (−26% reduction, *p* < 0.001). There were no significant differences in between intervention groups (Control vs. MD vs. MD + PA) for the change in steps or active kcals from usual activity across the time periods (*p* > 0.05) When comparing the effect of being assigned the PA component of the study, there was also no significant effects (Control + MD vs. MD + PA, *p* > 0.05).

### Influence of adiposity on physical activity levels

**Figure 3:**
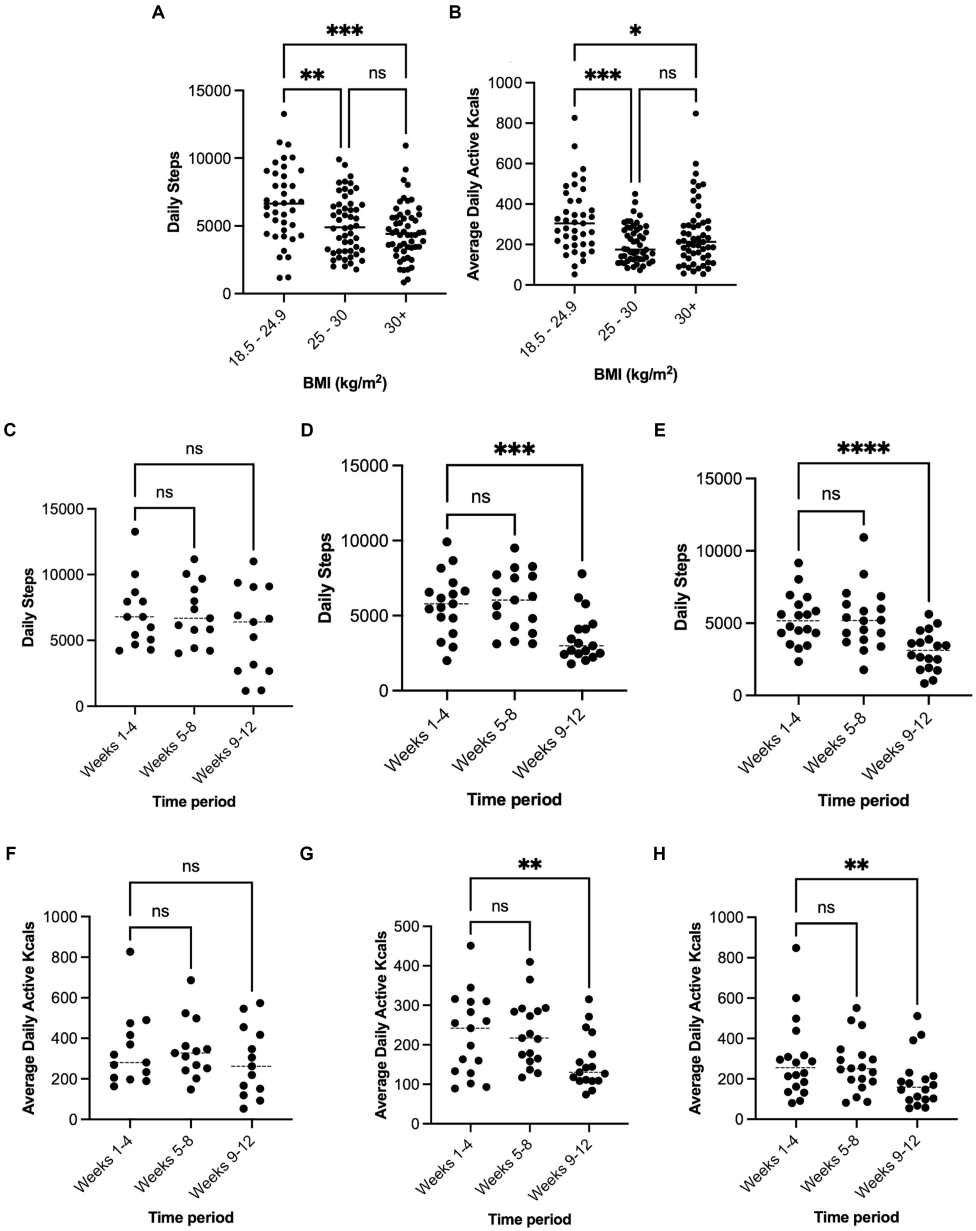
Mean average daily steps and active kcal expenditure across the 12-week period for each BMI category **(A,B)**. Panels **(C–E)** show average daily steps at stages of lockdown measures for 18.5–29.5, 25–30, and over 30 kg/m^2^ respectively. Panels **(F–H)** show average daily active kcal expenditure at stages of lockdown measures for 18.5–29.5, 25–30, and over 30 kg/m^2^ respectively. Significantly different to matched group, *p < 0.05, **p < 0.01, ***p < 0.001, ****p < 0.0001.

Throughout the 12-week period, the number of steps per day was significantly higher in participants with a healthy BMI (6,615 ± 2,783, *n* = 13) compared with participants who were overweight (5,094 ± 2,182, *p* = 0.006, *n* = 17) and those living with obesity (4,565 ± 2,030, *p* < 0.001, *n* = 18) with no difference between overweight and obese participants (*p* = 0.47). Patterns of change in active kcal expenditure across the 12 weeks of observation were similar with significantly higher values in participants with a healthy BMI (324.2 ± 165.7) compared with participants who were overweight (204.1 ± 91.76, *p* < 0.001) and those living with obesity (249.3 ± 159.3, *p* < 0.03) (Figure 3).

In both the usual activity period and lockdown periods, participants’ BMI correlated significantly, and inversely, with total steps per day [*R^2^* = 0.10, *F*(1, 46) 4.90, *p* = 0.03] and [*R^2^* = 0.17, *F*(1, 46) 9.31, *p* = 0.003], respectively. However, this relationship was not seen for active kcal expenditure (*p* > 0.05). In participants who were overweight and obese, the number of steps per day fell significantly from usual activity to lockdown (Overweight, 38% reduction to 3,574 ± 1,661, Obese, 41% reduction to 3,084 ± 1,347, *p* < 0.001), but did not differ between usual activity and the precautionary period (*p* > 0.05). In participants with a healthy BMI, the number of steps per day during lockdown tended to be lower (5,741 ± 3,332) but did not differ significantly from usual activity (7,008 ± 2,597, *p* = 0.11), or from during the precautionary period (7,097 ± 2,333). Active kcal expenditure in participants who were overweight and obese also fell between usual activity to lockdown (Overweight, 31% reduction to 156 ± 69, Obese, 36% reduction to 188 ± 129, *p* < 0.01), but did not differ in participants with a healthy BMI (341 ± 182 to 285 ± 173, *p* > 0.05).

### Differences in activity by age group

Throughout the 12 weeks, participants over 70 years (5,975 ± 2,626, *n* = 19) took significantly more steps per day compared with those under 70-year (4,853 ± 2,205, *p* = 0.007, *n* = 29) and had a greater daily active kcal expenditure (287 ± 158 vs. 232 ± 137, *p* = 0.003). However, participants in both age groups responded similarly to lockdown (*p* > 0.05).

### Differences in activity by deprivation score

Participants’ daily number of steps or active kcal expenditure during the usual activity phase did not differ by deprivation status (*p* > 0.05). Participants from both high and low deprivation areas experienced significantly reduced steps and active kcals in response to lockdown (*p* < 0.05). However, participants with a lower deprivation score (therefore higher deprivation) had a significantly greater reduction in daily steps (42% reduction, 6,039 ± 2,355 to 3,508 ± 17,08, *n* = 25) compared with those with higher deprivation scores (23% reduction, 5,742 ± 2034 to 4,487 ± 2,894, *p* = 0.03, *n* = 23). This difference was not seen for active kcal expenditure (*p* > 0.05).

### Influence of baseline (usual activity period) PA levels

There was no relationship between levels of PA during usual activity, expressed as both daily steps and active kcal expenditure, and change in PA in response to lockdown (*p* > 0.05).

### Qualitative data synthesis

The quotes used illustrate participants’ experiences during lockdown, and the perceived impact of COVID-19 lockdown on their PA. Participants highlight the barriers affecting their ability to engage in PA, and share experiences where there were some unexpected positive impacts that lockdown had on their PA.

### Barriers to physical activity

During the COVID-19 pandemic, some participants were shielding, either for themselves or in caring for others and were ‘nervous about going out’ and wary as other people did not keep their distance from them. After a walk, one participant reflected:

*You find some of the younger joggers and people walking don’t necessarily try and maintain their distance from you* (Participant 3, Focus group 6, W1, Newcastle).

Several activities were put on pause during lockdown, including community activities such as parkrun, and activities such as walking groups had ‘broken down’ due to lockdown and the temporary closure of gym facilities, preventing swimming and exercise classes. Participants felt ‘disappointed’ or ‘disheartened’ by these stops to their activities.

Whilst PA plans were derailed, some participants described taking on alternative activities such as walking by themselves, gardening, or cycling; activities that could be completed outside, independently. These activities were often seen as subpar because participants felt they did not raise their heart rate as much as other planned activities. One participant explained:

*I had loads of things planned. I had swimming. I had booked to do the Park Run.… of course it all stopped so literally I’ve either cycled or walked* (Participant 2, focus group 1, W2, Birmingham).

### Increasing physical activity

If not impacted by shielding, or barriers such as injury or illness, a few participants described lockdown as a time where they increased their PA levels. One participant described:

*I have increased my activity level pretty much simply because of lockdown really. Just lots more walking, more conscious of exercise* (Participant 3, focus group 1, W1, Birmingham).

Although most participants were retired, those experiencing a change in working arrangements, and flexibility in working hours, perceived impacts on PA levels. Increasing PA was generally weather dependent, as described by participants who walked, and one cyclist described themselves as a ‘fair weather cyclist’. Another participant reflected on the impacts of changes to work due to lockdown, and the better weather:

*I can honestly say that I’ve done more walking and cycling, which are the two main things I would do anyway…When I was in the office, I would walk out at lunchtime whenever I could, but I would only do 20 min max so 10 min out, 10 min back. With lockdown, once I started working from home, because I had more flexible hours, I could fit it in, we walked every day. We walked out every lunchtime and I recorded in on MapMyWalk every time we went out and I know for a fact that we did virtually in the 100 odd days I think we walked you know probably 95% of those days for at least a half hour at lunchtime and that would be a mile and a half and it would be at a fair pace*.

*The other side is I have my bike and I know I’ve averaged, since lockdown, since the weather got better, 100 miles a month which I’m quite pleased with that. It’s gone away the last few weeks simply because of the weather, you know the wind and the rain so… actually through lockdown the main part from March onwards those first two or three months the weather was good so it was conducive to actually getting out and about anyway* (Participant 1, focus group 6, W1, Newcastle).

Lockdown prompted participants to value their time outside, and some participants described it as their only time spent out of the house. One participant explained the change in their behaviour:

*There’s been very few days where I haven’t actually gone out for a walk. In March, April, May when we really were stuck in it was nice to go for a walk* (Participant 4, focus group 1, W1, Birmingham).

## Discussion

The present study assessed the impact of lockdown measures enforced in the United Kingdom in response to the COVID-19 pandemic amongst a highly characterised cohort of older adults at risk of cardiovascular disease. On average, lockdown was accompanied by a significant reduction (28%) in objectively measured PA (assessed by daily step counts and active kcal expenditure), which suggests a significant shift in behaviours across this period. This corresponded to a reduced step count of around 2,000 steps/day on average which is likely to impact health outcomes, especially if reduced activity is prolonged on return from the pandemic. In fact, even a difference of 1,000 steps per day is inversely associated will altered risk of all-cause mortality and CVD risk (41, 42).

Perhaps most strikingly, the greatest reduction in PA levels was in participants who had a BMI in the overweight or obese category compared with individuals with a healthy BMI who maintained pre-pandemic PA levels. Since obesity is a major risk factor for cardiometabolic disease, reductions in PA of this magnitude are likely to have severe negative implications for both short-term and long-term health. Qualitative data synthesis highlighted the impact of changing access to facilities as a major barrier to PA. Although participants wished to engage in higher levels of PA, the social and physical opportunity to participate in PA was restricted (4). This was particularly evident in participants perceiving themselves to be at higher risk of COVID-19.

The measures implemented by the United Kingdom government during such lockdowns to reduce risk of infection such as self-isolation, social distancing, and restricted access to group activities and leisure activity centres, could reasonably explain the observed reduction in PA (43). This finding is in line with self-reported data showing reduced PA and increased sedentary time across several populations with differing demographics (19, 22, 44). Our participants highlighted changes in their PA behaviour during lockdown, particularly linked to decreased structured activities, e.g., enforced closure of fitness facilities, and restricted outdoor activities. Three factors have been identified as necessary for the performance of a specified volitional behaviour, e.g., PA: the skills necessary to perform the behaviour, a strong intention to perform the behaviour, and no environmental constraints that make it impossible to perform the behaviour (4). Fundamentally, the third factor necessary for PA was impeded. Linked to this, the context stability can predict behaviour, i.e., a more stable physical context predicts more moderate-vigorous activity (45). Alternatively, a more stable social context for sitting and more variable times of sitting predict more sedentary behaviour. Context instability, induced by lockdown measures created changes in daily habits, or disruption to planned positive PA action.

Despite these challenges, it is important to note that some older adults found alternative ways to continue being physically active. Participants described taking alternative PA action, including engaging with individual, outside activities, mostly walking and gardening, with some participants cycling. For some, this was led to a decrease in their PA intensity. Noteworthy, for some participants (those not shielding, and those who still worked), the lockdown provided an opportunity to engage with PA behaviours through work flexibility, or through the heightened importance placed on going outside as their only time spent out of the house. Evidence during the early lockdown periods outside of the United Kingdom have also shown an increase in physical activity for certain individuals. Physical activity for individuals who played golf in Finland increased and was linked to a high quality of life (46). This raises two important considerations. First is that there may be different PA responses to periods of restriction depending on seasonality, whereby PA will be higher in warmer months. Secondly, restrictions related to COVID-19 varied significantly between countries and future research should compare how differing measures impacted PA and overall wellbeing to identify the ‘most effective’ model to minimise transmission whilst promoting good physical health.

Negative effects of lockdown on eating and PA behaviours, as well as increased perception of barriers to weight management, in people with an elevated BMI have been reported previously (19, 22). The risks of implementing lockdown measures have also been highlighted for older adults as increased weight gain and reduced PA (47). However, in this study, we found that older adults tended to engage in a greater number of daily steps across the 12-week study period and responded similarly in terms of PA reduction to younger adults. This may be explained by the recruitment of participants who had a QRISK score ≥ 10, as age is a major driver of the score, our older adults may reflect a healthier population relative to other comorbidities (higher BMI, diabetic, and medication). Our qualitative data indicated that this may also be due to the flexibility of working from home, or not having formal work commitments for retired participants and the importance participants placed on the time spent outside during this lockdown period. Similar PA findings have also been reported in cross-sectional analysis suggesting that increased age alone, was not associated with changes in PA in response to the United Kingdom lockdown (47, 48). There is also some evidence that older adults may have increased PA as lockdown measures eased (49), but the present study did not quantify PA levels after the lockdown period when government restrictions were eased.

The impact of the COVID-19 pandemic on health disparities is a particular social challenge as those with poorer health, increased vulnerability to COVID-19, worse mental health pre-pandemic and living in socio-economically deprived areas had significantly worse outcomes (50). These inequalities are amplified in black and minority ethnic groups especially those from less advantaged socio-economic backgrounds (51). However, evidence from other research has suggested that deprivation was not a determinant of PA during the pandemic (51). In fact, changes in PA were more likely related to pre pandemic activity levels, with those who were most active prior to restrictions seeing the greatest reduction in PA during lockdown (52). However, we did not find this association in our study. Future research should consider the potential differential impact of government lockdown measures across diverse groups to improve the United Kingdom’s response to potential future pandemics. Developing targeted strategies that protect high-risk populations from the risks of communicable disease whilst maintaining PA and health status is of importance.

A key strength of our study is the objective PA data collection over 12-continuous weeks, which aligns with public health research recommendations for objective and comprehensive evaluation of health promotion programmes (53). In addition, by combining quantitative methods investigating modulators of PA changes during lockdown, alongside qualitative data, we provide more detailed information how government-imposed restrictions designed to address the COVID-19 pandemic influenced PA. However, this study has several limitations. An important limitation is that we are unable to tell what type of activity was reduced by lockdown measures (i.e., exercise or non-exercise PA). This is important as it impacts the interpretation of the findings and our understanding of measures that could be put in place to mitigate physical inactivity in future crisis scenarios. For example, older people may be able to maintain PA levels via non-exercise PA (walking, gardening, cycling etc.) if access to facilities for exercise (structured and repetitive PA) is restricted. In addition, the use of wrist-based PA monitors has limitations. Tracking daily steps can be accurately measured usually however, energy expenditure calculations using optical heart rate are less valid and variation between devices is significant (54, 55). To increase data reliability, we collected continuous daily data and averaged activity over 4-week periods.

Overall, this study showed that the lockdown measures enforced in response to the COVID-19 pandemic impacted PA behaviours of older individuals at risk of cardiovascular disease and participants reduced PA levels as measured by both step count and energy expenditure. However, BMI appeared to be a significant predictor of PA levels in response to lockdown measures, with higher BMI linked to lower PA during the lockdown period. For most participants, the lockdown measures enforced in response to the COVID-19 pandemic were a major barrier to PA. Supporting obese and overweight older adults in maintaining PA when there are restrictions to facilities and in the event of any future lockdown will require a multi-faceted approach. This will need to consider ways to promote and encourage engagement with accessible PA, providing resources and guidance, and supported by appropriate behaviour change approaches, e.g., monitoring and goal setting.

## Data availability statement

The raw data supporting the conclusions of this article will be made available by the authors, without undue reservation.

## Ethics statement

Ethical approval for the study was given by the National Research Ethics Committee Northern Ireland (18/NI/0191). The authors confirm that all necessary patient/participant consent has been obtained and the appropriate institutional forms have been archived, and that any patient/participant/sample identifiers included were not known to anyone (e.g., hospital staff, patients, or participants themselves) outside the research group so cannot be used to identify individuals. The studies were conducted in accordance with the local legislation and institutional requirements. The participants provided their written informed consent to participate in this study.

## Author contributions

RE: Formal analysis, Investigation, Writing – original draft, Writing – review & editing. SJ: Data curation, Methodology, Writing – original draft. SH: Formal analysis, Investigation, Methodology, Writing – original draft. OS: Conceptualization, Data curation, Investigation, Methodology, Writing – original draft, Writing – review & editing. AJ: Methodology, Writing – review & editing. RG: Data curation, Investigation, Writing – review & editing. MS: Conceptualization, Resources, Supervision, Writing – review & editing. MH: Conceptualization, Data curation, Funding acquisition, Investigation, Supervision, Writing – review & editing. WH: Conceptualization, Investigation, Supervision, Writing – review & editing. JM: Conceptualization, Funding acquisition, Methodology, Supervision, Writing – review & editing. A-MM: Conceptualization, Funding acquisition, Project administration, Supervision, Writing – review & editing. SA: Funding acquisition, Resources, Supervision, Writing – original draft, Writing – review & editing.

## Footnotes

^1^ http://imd-by-postcode.opendatacommunities.org/imd/2019
